# Acetolactate synthase regulatory subunits play divergent and overlapping roles in branched-chain amino acid synthesis and Arabidopsis development

**DOI:** 10.1186/s12870-017-1022-6

**Published:** 2017-04-07

**Authors:** Mohammad H. Dezfulian, Curtis Foreman, Espanta Jalili, Mrinal Pal, Rajdeep K. Dhaliwal, Don Karl A. Roberto, Kathleen M. Imre, Susanne E. Kohalmi, William L. Crosby

**Affiliations:** 1 Department of Biological Sciences, University of Windsor, Windsor, ON Canada; 2 Department of Biochemistry and Molecular Biology, Michigan State University, Lansing, MI USA; 3 Department of Biology, University of Western Ontario, London, ON Canada; 4 Present address: Department of Genetics, Harvard Medical School, Boston, MA 02115 USA

**Keywords:** Branched-chain amino acids, Acetolactate synthase, ALS, AHAS, Valine, Isoleucine, Leucine

## Abstract

**Background:**

Branched-chain amino acids (BCAAs) are synthesized by plants, fungi, bacteria, and archaea with plants being the major source of these amino acids in animal diets*.* Acetolactate synthase (ALS) is the first enzyme in the BCAA synthesis pathway. Although the functional contribution of ALS to BCAA biosynthesis has been extensively characterized, a comprehensive understanding of the regulation of this pathway at the molecular level is still lacking.

**Results:**

To characterize the regulatory processes governing ALS activity we utilized several complementary approaches. Using the ALS catalytic protein subunit as bait we performed a yeast two-hybrid (Y2H) screen which resulted in the identification of a set of interacting proteins, two of which (denoted as ALS-INTERACTING PROTEIN1 and 3 [AIP1 and AIP3, respectively]) were found to be evolutionarily conserved orthologues of bacterial feedback-regulatory proteins and therefore implicated in the regulation of ALS activity. To investigate the molecular role AIPs might play in BCAA synthesis in *Arabidopsis thaliana*, we examined the functional contribution of *aip1* and *aip3* knockout alleles to plant patterning and development and BCAA synthesis under various growth conditions. Loss-of-function genetic backgrounds involving these two genes exhibited differential aberrant growth responses in valine-, isoleucine-, and sodium chloride-supplemented media. While BCAA synthesis is believed to be localized to the chloroplast, both AIP1 and AIP3 were found to localize to the peroxisome in addition to the chloroplast. Analysis of free amino acid pools in the mutant backgrounds revealed that they differ in the absolute amount of individual BCAAs accumulated and exhibit elevated levels of BCAAs in leaf tissues. Despite the phenotypic differences observed in *aip1* and *aip3* backgrounds, functional redundancy between these loci was suggested by the finding that *aip1/aip3* double knockout mutants are severely developmentally compromised.

**Conclusions:**

Taken together the data suggests that the two regulatory proteins, in conjunction with ALS, have overlapping but distinct functions in BCAA synthesis, and also play a role in pathways unrelated to BCAA synthesis such as sodium-ion homeostasis, extending to broader aspects of patterning and development.

**Electronic supplementary material:**

The online version of this article (doi:10.1186/s12870-017-1022-6) contains supplementary material, which is available to authorized users.

## Background

The BCAAs leucine, isoleucine, and valine are synthesized by plants, fungi, bacteria, and archaea [1]. The de novo synthesis of BCAAs has been an historical object of attention for two main reasons: this pathway is a known target for at least five independent classes of inhibitory herbicides [2] and, secondly, animals lack the necessary genes encoding enzymes for BCAA synthesis thus requiring that this class of essential amino acids be obtained via dietary intake. These features have stimulated interest in the genetic manipulation of plant BCAA metabolism both for the generation of herbicide-tolerant crops as well as enhanced nutritional value through increased levels of storage BCAAs [3, 4]. Despite the importance of the BCAA synthesis pathway in plants, knowledge pertaining to regulation of this pathway has mostly originated from studies in non-photosynthetic bacteria and yeast [5, 6].

Valine (Val) and isoleucine (Ile) are synthesized via two parallel pathways involving four common enzymes, which catalyze different products in the presence of different substrates. The first common enzyme in the pathway, acetolactate synthase (ALS, EC 2.2.1.6; also known as acetohydroxyacid synthase, AHAS), utilizes either two pyruvate molecules for the formation of acetolactate as a precursor to Val and leucine (Leu) biosynthesis, or one pyruvate plus one α-ketobutyrate substrate molecule to generate acetohydroxybutyrate as a precursor to Ile biosynthesis [7]. The de novo BCAA synthesis pathway is thought to be negatively regulated through feedback inhibition by their final BCAA products acting at the level of multiple enzymes [8]. While ALS enzymatic activity has been shown to be potently feedback-inhibited by Val, Ile, and Leu in vitro [9], the current literature lacks an in vivo-based assessment of this proposed model.

The ALS holoenzyme is composed of distinct catalytic and regulatory subunits in an α_4_β_4_ quaternary configuration in *Arabidopsis thaliana* [5]. The incorporation of regulatory subunits to the catalytic quaternary complex is hypothesized to stabilize the ALS complex, resulting in an almost five-fold enhancement of ALS catalytic activity [10]. The regulatory subunit is also responsible for end-point inhibition of ALS catalytic activity by interaction with a highly-conserved ACT domain [6]. A distinguishing feature among the bacterial and plant regulatory subunits is the larger size of the plant regulatory subunits, where the plant protein is composed of a pair of tandem repeats of a single orthologous prokaryotic regulatory subunit domain. The two putative regulatory subunits identified in Arabidopsis each contain two ACT domains, thus raising the possibility that each ACT domain could serve as a functionally distinct binding domain for BCAA-mediated feedback inhibition [6].

Plant genomes commonly contain multiple genes known or predicted to encode ALS regulatory subunits, including two putative regulatory ALS subunit genes found in the relatively small genome of Arabidopsis. The high primary sequence similarity observed between the two regulatory subunit proteins in Arabidopsis is suggestive of functional redundancy between these two proteins. However, the suggestion of simple functional redundancy is complicated by two observations. First, recent forward genetic screens in Arabidopsis aimed at identifying mutants resistant to the growth-inhibitory effects of BCAAs revealed several alleles at a single genetic locus encoding only one of the two known ALS regulatory subunits [3]. Secondly, although both regulatory subunit proteins are highly similar in their overall primary amino acid sequence, they differ significantly within the first 85 N-terminal amino acids, as well as within the second repeated ACT domain as the proposed Val binding site [6]. Given that gene duplication provides evolutionary ‘raw material’ for functional diversification [11], it is possible that a level of sub-functionalization has evolved among the two regulatory subunits, with the corresponding possibility of a novel framework for the regulation of BCAA synthesis in plants.

Although the ALS holoenzyme has been an object of study in the context of BCAA synthesis, relatively little is known about the regulatory mechanisms governing its activity. For instance, despite the essential requirement of the ALS catalytic subunit for plant growth and development, no other interacting partners apart from the regulatory subunits have been reported thus far. Furthermore, it is not known whether the regulatory or the catalytic subunits of ALS functionally contribute to other aspects of plant growth and development outside the context of BCAA synthesis. To explore the regulatory mechanisms controlling BCAA biosynthesis in plants, we have undertaken a set of complimentary functional studies that led to the identification of multiple novel ALS binding partners. Furthermore, we provide genetic and biochemical evidence suggesting that the putative regulatory subunits AIP1 and AIP3 impart overlapping yet divergent functions essential for growth and development in Arabidopsis. These divergent functions are not limited to BCAA biosynthesis, suggesting that these proteins have acquired additional and novel functional roles during the course of plant evolution.

## Results

### Y2H screen identifies novel and existing protein interactors of ALS catalytic subunit

**Fig. 1:**
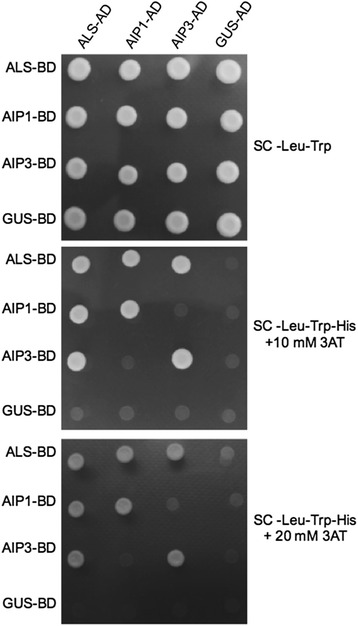
ALS and AIP interaction analysis using yeast two-hybrid. Assessment of ALS-AIP and AIP protein interaction using Y2H assays. Colonies expressing the designated constructs and grown on –Leu –Trp + His plates (*top* panel) and test plates containing the indicated concentrations of 3-AT in the absence of Leu/Trp/His (*bottom* panels) were imaged. Strong homo-oligomers are observed for both ALS and AIPs. Heterodimers are observed between ALS and AIPs but not between AIP1 and AIP3. GUS CDS was used as a negative control

To gain a better understanding of the ALS protein interactome, we conducted a yeast two-hybrid (Y2H) screen using the ALS catalytic subunit as bait, leading to the identification of several proteins. Following the sequencing of the corresponding fusion cDNA sequences involved, the deduced protein sequence of the identified ALS interactors fell into eight classes (Table 1) and were denoted as ALS-interacting proteins (AIPs). All interacting pairs were again independently verified using Y2H (data not shown). Two out of the eight classes, *AIP1* (AT2G31810) and *AIP3/VAT1* (AT5G16290), showed high deduced protein similarity to prokaryotic and eukaryotic regulatory ALS subunits (Additional file 1: Figure S1), suggesting that both these putative regulatory subunits associate with the ALS catalytic subunit *in planta*. In the absence of information pertaining to the binding affinities of the regulatory subunits with the catalytic subunit, we assessed the qualitative binding stability of both proteins using the indirect measure of Y2H signal intensity in yeast. No detectable difference in subunit-binding stability was observed among the catalytic subunit and the two regulatory proteins (Fig. 1).

**Table 1:** ALS Interacting proteins identified from the Y2H screen

Gene name	Locus	Localization	Reference
ALS Small Regulatory Subunit (AIP1)	AT2G31810	Peroxisome, Plastid	This study, MS/MS
Protein of unknown function (DUF567)	AT5G01750	Plastid, Plasma Membrane	Bioinformatics
ALS Small Regulatory Subunit (AIP3)	AT5G16290	Peroxisome, Plastid	This study, MS/MS, Bioinformatics
FAD/NAD(P)-binding oxidoreductase family protein	AT4G38540	Plastid, Plasma Membrane	MS/MS, Bioinformatics
CHLOROPLAST CHAPERONIN 10 (CPN10)	AT2G44650	Plastid, Mitochondria	Immunohistochemistry
PYRIDOXINE BIOSYNTHESIS 1.1 (PDX1.1)	AT2G38230	Mitochondria, Cytosol, Plastid	BiFC, Bioinformatics, GFP, MS/MS
RING/U-Box superfamily protein	AT2G21500	Plastid, Mitochondria, Plasma Membrane, Peroxisome	Bioinformatics, MS/MS
Protein of unknown function – Glycine Rich	AT3G23450	Plastid, Plasma Membrane, Endoplasmic Reticulum	Bioinformatics

Subcellular localization data was acquired from the July 2016 version of the SUBAcon resource [14]

Solution-based structural analyses have revealed that the ALS holoenzyme exists in an oligomeric state. Furthermore, experimental data have indicated that the formation of dimeric catalytic subunits is likely essential for active site formation [12]. To assess the capacity of the identified regulatory subunits to self-interact, we performed a series of Y2H experiments involving pair-wise combinations of all interacting subunits thus far identified. These in vivo results confirmed the reported in vitro homo-oligomerization of the ALS catalytic subunit [13], and showed that both AIP1 and AIP3 catalytic subunits are also able to homo-oligomerize (Fig. 1).

Despite the high sequence similarity between AIP1 and AIP3, and given that both proteins can independently homo-oligomerize, we were unable to detect hetero-oligomerization between these two proteins (Fig. 1). The results suggest that the ALS holoenzyme may exist as a population of two independent quaternary complexes, each involving the catalytic subunit in complex with either AIP1 or AIP3 regulatory proteins.

In addition to AIP1 and AIP3, all the ALS-interacting proteins identified by our Y2H studies, have either been experimentally verified to localize to the chloroplast or are computationally predicated to harbor a chloroplast localization transit signal peptide (Table 1) [14]. The plastid co-localization of ALS and the novel interacting partners identified here is further validation of the biological relevance of these interactions.

### *AIP1* and *AIP3* exhibit divergent expression patterns during development

**Fig. 2:**
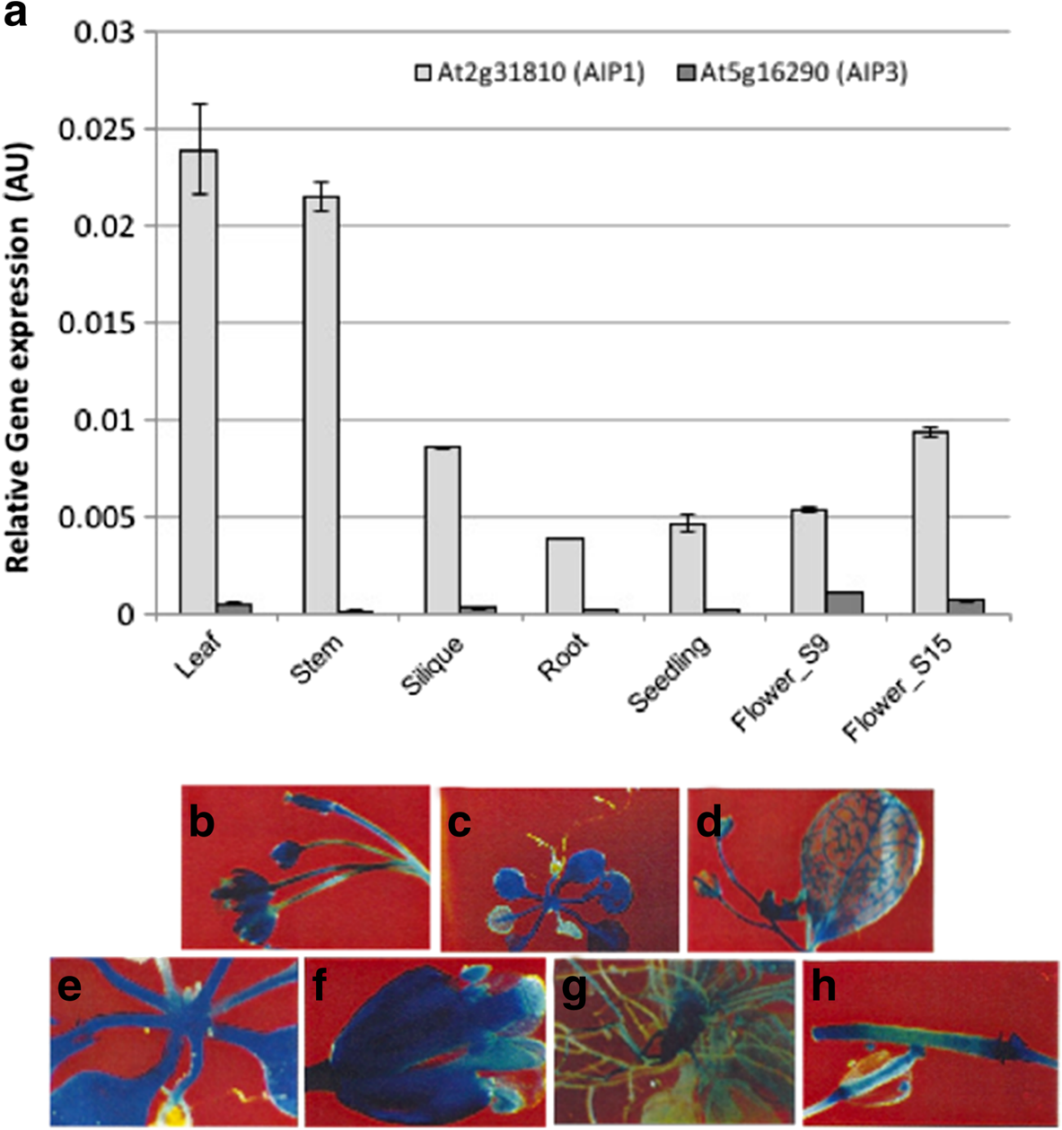
*AIP1* and *AIP3* have divergent expression patterns. **a** RNA from the indicated organs was isolated and used to assess *AIP1* and *AIP3* transcript levels as described in the methods. Organs examined were 4–5-day-old seedlings, stage 5 leaves, green stem, green siliques, stage 9 flowers, and stage 15 flowers. Relative gene expression profiles ± SE after *ACTIN2* normalization are depicted. Data presented is representative of two independent biological replicas each with three samples repetitions ± SE. **b**-**h** GUS representative expression profiles for AIP1 (750 bp of 5′ putative promoter) promoter-driven GUS transgenic lines

The expression pattern of one the regulatory subunits—*AIP3/VAT1*—was recently investigated *in planta* using transgenic reporter-gene fusion constructs [3]. However, a quantitative expression analysis of transcript abundance for both regulatory subunits in different tissues and at different stages of development has not been described. We elected to undertake a quantitative study to provide the desired precision needed for higher-resolution comparison of regulatory subunit expression profiles. Such studies could be important for defining the extent of expression overlap among subunits, and for elucidating their potential to participate in the formation of ALS complexes across developmental time and space. Accordingly, a qRT-PCR analysis of transcript abundance was undertaken for both *AIP1* and *AIP3* in select tissues taken from plants at different stages of development. Transcript abundance was quantified in cDNA preparations generated from total RNA fractions prepared from rosette leaves of plants prior to stage 5.2, roots of 7-day-old seedlings, green stems (1^st^ and 2^nd^ internodes of bolted plants), green siliques with seeds (late heart to mid-torpedo embryo), whole 5-day-old seedlings, advanced stage 9 whole flowers (petal primordia stalked at base), 19–23-day-old plants, stage 15 whole flowers (stigma extends above long anther) and from 21 to 23-day-old plants (Fig. 2a). Although we were able to detect transcripts in all the tissues assessed, there was a marked difference between the abundance of the two transcripts where *AIP1* mRNA was relatively more abundant in all tissues examined. To further investigate the expression pattern of *AIP1*and *AIP3*, transgenic plants expressing an *AIP1* promoter-driven β-glucuronidase (GUS) reporter gene were generated and GUS enzyme activity was examined in ten independent transgenic lines. All transgenic lines exhibited GUS expression patterns that correlated with transcript abundance profiles defined by the qRT-PCR analyses (Fig. 2b-h). The observed ratio of *AIP1* to *AIP3/VAT1* transcript abundance was strikingly divergent across developmental time and space. This finding was especially evident when comparing two different stages of flower development, where a drastic decrease in *AIP1* expression was observed, concomitant with a marked increase (at least three-fold) for *AIP3/VAT1*. This observation suggests that both ALS/AIP holoenzymes can simultaneously exist in the proteome, where the ratio of ALS/AIP1 to ALS/AIP3 holoenzymes may play differential roles during plant growth and development.

### ALS regulatory subunits localize to both the peroxisome and chloroplast

**Fig. 3:**
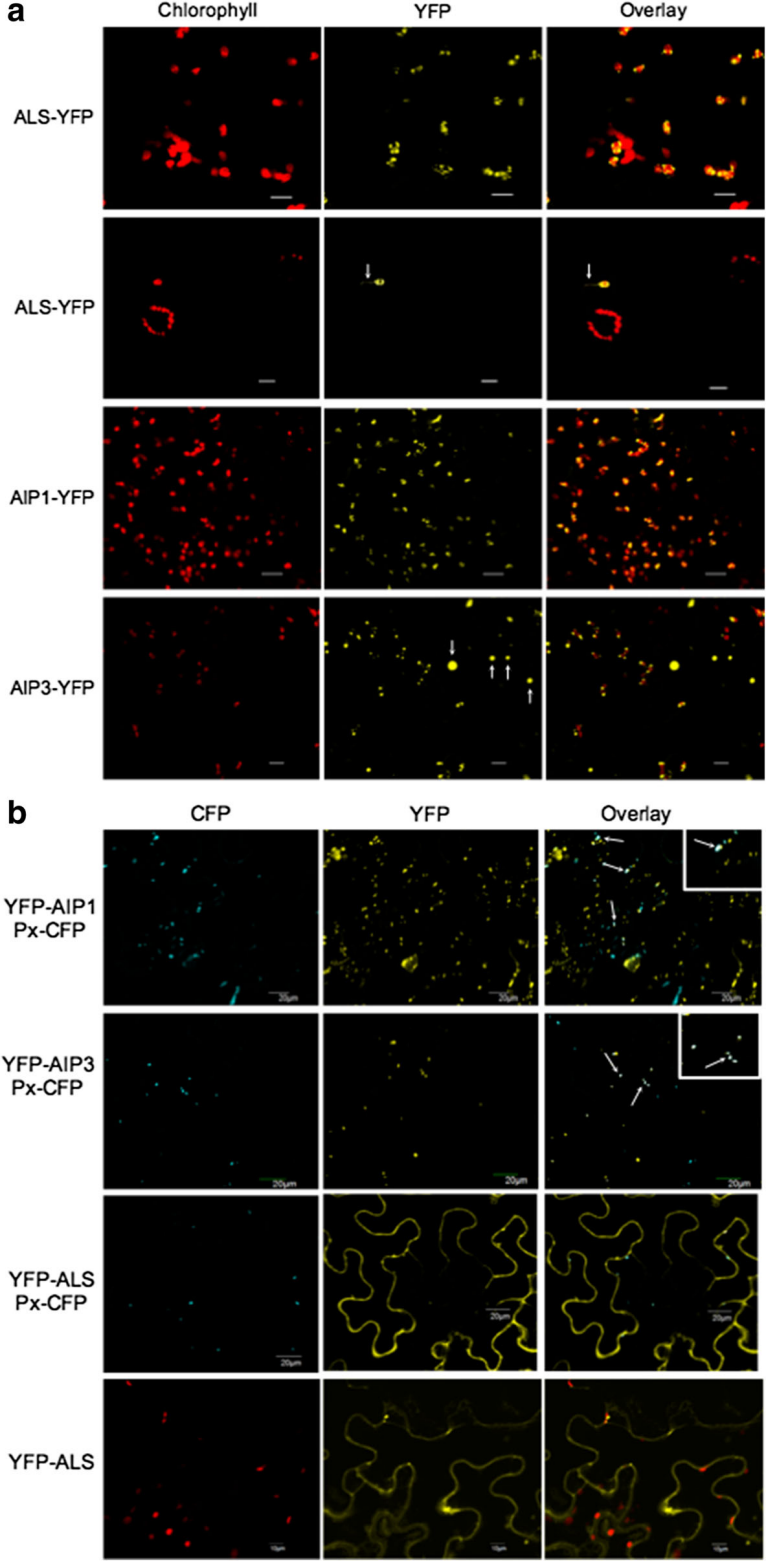
AIP1 and AIP3 localize to both the chloroplast and peroxisome. **a** Subcellular localization of C-terminal YFP-tagged fusions of ALS, AIP1, and AIP3 in *N. benthamiana*. ALS-, AIP1-, and AIP3-YFP were expressed in Nicotiana leaves and analyzed by confocal microscopy. The localization of the AIPs and ALS are visualized by yellow fluorescence. Red fluorescence: excited chlorophyll emission to mark plastid location. YFP-ALS arrowheads: stromal localization of C-terminal YFP-tagged fusion, YFP-ALS. AIP3-YFP arrowheads: localization of AIP3-YFP outside of chloroplasts. Scale Bar, 20 μm (**b**) Subcellular localization of ALS, AIP1, AIP3, N-terminal YFP tagged fusions in Nicotiana leaves along with a peroxisomal marker (Px-CFP). Red fluorescence: excited chlorophyll emission to mark plastid location. Arrowheads: co-localization of Px-CFP with AIP1-YFP and AIP3-YFP

AIP1 and AIP3 proteins share ~80% identity at the deduced amino acid level (Additional file 1: Figure S2). The two deduced protein sequences were most divergent within the N-terminal 85 amino acids where sub-cellular localization signaling peptides typically reside. Although it has been well established that the initial catalytic reaction in BCAA synthesis is mediated by ALS enzymes in the chloroplast, no experimental data currently exist to localize individual ALS regulatory subunits to the chloroplast. We therefore evaluated the sub-cellular localization profile of ALS catalytic and regulatory subunits expressed as functional fusions with the auto-fluorescent yellow fluorescent protein (YFP). Whereas the C-terminal-tagged YFP-fusion of both regulatory and catalytic subunits localized to the plastid, the AIP3-YFP fusion protein also localized as dense signal-foci of varying sizes outside of the plastid (white arrows; Fig. 3a). The observed pattern resembled that of peroxisome localization, which led us to ask whether AIP3 also localizes to the peroxisome. We therefore assessed the co-localization of AIP3-YFP with a CFP-tagged peroxisome (Px-CFP) marker protein [15]. The results revealed that the YFP and CFP signals co-localize, suggesting a dual localization for AIP3-YFP to both the plastid and the peroxisome (Fig. 3b). To assess whether the potential masking of the otherwise exposed N-terminal plastid localization signal would have an effect on the peroxisomal localization of these proteins, we generated the reciprocal set of N-terminal YFP fusion proteins and assessed their sub-cellular localization. All three N-terminal YFP-tagged proteins were found to be mostly excluded from the plastid, with ALS-YFP accumulating only in the cytoplasm (Fig. 3b). However, both YFP-AIP1 and YFP-AIP3 fusion proteins were not only excluded from the chloroplast but also readily co-localized with the Px-CFP peroxisomal marker (Fig. 3b). Although both AIP1 and AIP3 regulatory proteins possess an identifiable N-terminal plastid localization domain [16], neither protein was found to contain a canonical PTS1 or PTS2 peroxisomal localization signal [17]. However, upon closer analysis, potential “cryptic” PTS1 motifs are found in the C-terminal region of both AIP1 (amino acid sequence S-K-Y) and AIP3 (S-T-Y) proteins, which may be important given that the SKY motif was recently shown to confer peroxisomal localization to a typically plastid-localized glucose-6-phosphate dehydrogenase protein [18]. The results suggest that both AIP1 and AIP3 regulatory subunits localize to the chloroplast as well as the peroxisome. Given the presence of a cryptic PTS1 motif, together with C-terminal peroxisomal targeting following masking of the plastid transit peptide, the structural and experimental results support the suggestion of a dual subcellular localization of these two proteins that in itself may be an object of regulation during development [19].

### Functional assessment of *AIP1* and *AIP3*

**Fig. 4:**
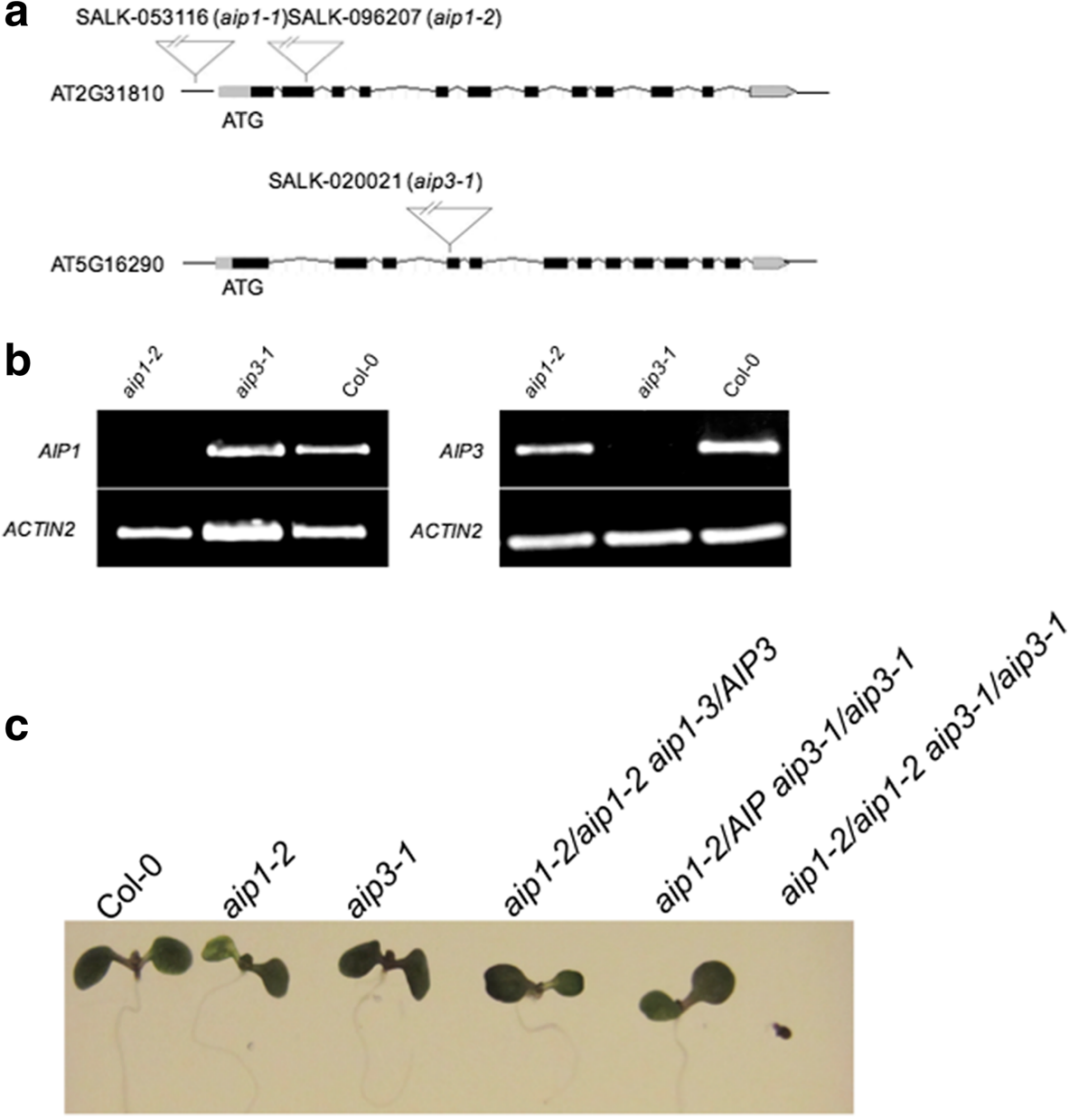
*AIP1* and *AIP3* are essential for Arabidopsis growth and development. **a** Schematic representation of the T-DNA insertion *aip1-1*, *aip1-2*, and *aip3-1* mutants. **b** Assessment of expression of *AIP1* and *AIP3* in *aip1-2* and *aip3-1* knockout lines when using RT-PCR. **c** Phenotypes of 9-day-old wild type, single and double *aip* knockout mutants. The *aip1-2/aip3-1* double knockout mutant, *Col*-*0* and the single *aip* knockout mutants grown on control media (0.5× MS), only the double *aip* knockout mutants exhibit a synthetic lethal phenotype, then fail to develop adult organs followed by chlorosis and death

To explore the functional roles of *AIP1* and *AIP3* genes in Arabidopsis patterning and development, we exploited a reverse genetics approach involving the analysis of plants carrying single as well as multiple loss-of-function alleles in these two genes. Plant lines homozygous for inactivating T-DNA insertions in *AIP1* and *AIP3* were identified from the SIGnAL resource [20]. The mutant alleles were designated as *aip1-1*, *aip1-2*, and *aip3-1*, and the precise location of each insertion was verified by sequence analysis (Fig. 4a). Among the three lines assessed, two mutant lines were confirmed as inactivating knockout alleles as evidenced by qRT-PCR analysis, since no detectable levels of *AIP1* and *AIP3* transcripts were observed in the homozygous *aip1-2* and *aip3-1* mutant lines, respectively (Fig. 4b). Thus, *aip1-2* and *aip3-1* lines harbor null alleles of AT2G31810 and AT5G16290, respectively. In the case of the *aip1-1* mutant, the T-DNA insertion was found to reside in the presumed promoter region and we were unable to detect any difference in the *AIP1* transcript abundance levels between wild-type and plants homozygous for this T-DNA insertion (data not shown).

Plants homozygous for the *aip1-2* or *aip3-1* mutant alleles were backcrossed for three to four generations and used for phenotypic analysis. These mutants were morphologically similar to wild-type plants under normal growth conditions and developed normal roots, leaves, shoots, and flowers. Furthermore, the single-mutant lines did not exhibit any significant difference in hypocotyl elongation or germination rate (data not shown).

To test for possible functional redundancy between *AIP1* and *AIP3*, we attempted to generate *aip1/aip3* double mutant lines (*aip1-2/aip1-2 aip3-1/aip3-1*). While we were able to readily identify *aip1-2/AIP1 aip3-1/aip3-1* and *aip1-2/aip1-2 AIP3/aip3-1* genotypes among the F2 progeny derived from a cross between the *aip1* and *aip3* single mutants, we were unable to identify double mutant plants among the F3 progeny seeds. After numerous unsuccessful attempts to identify a double-mutant line among healthy plants, we reasoned that the double mutant was either lethal, or that double-mutant progeny were severely developmentally compromised. Accordingly, we analyzed segregating progeny for stunted plants or those that arrested early in development. From these surveys, we were able to identify double homozygous mutant seedlings, but only among the segregating progeny of the *aip1-2/aip1-2 AIP3/aip3-1* parental background. The double homozygous mutant lines identified became chlorotic and died before the first two true leaves emerged at growth stage 1.02 (Fig. 4c). Seeds collected from the *aip1-2/aip1-2 AIP3/aip3-1* mutant backgrounds all germinated and grew into normal plants. This finding prompted us to investigate the seed-set and silique morphology phenotypes among the various mutant backgrounds. This analysis revealed that the *aip1/AIP1 aip3/aip3* line exhibited a 40% reduction in the production of viable seed compared to all other genetic lines examined (Additional file 1: Figure S3B). Aborted seeds could be observed as ovule ‘vacancies’ in unopened siliques; in particular, in the *aip1/AIP1 aip3/aip3* mutant background (Additional file 1: Figure S3A). As part of the gross phenotypic analysis, later stages of development were also characterized, but no differences were observed in growth phenotypes between the various knockout mutants compared and wild-type plants.

### *AIP* mutants respond differently to exogenously applied branched-chain amino acids

**Fig. 5:**
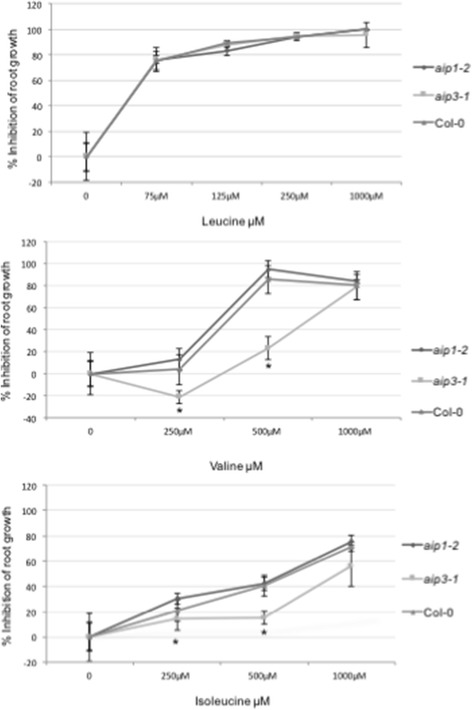
AIP3 plays a dominant role during BCAA growth inhibition. Primary root inhibition was measured in *aip* mutants and wild type plants on BCAA-supplemented media. Percent primary root inhibition of 5-day-old seedlings grown on Leu-, Val-, and Ile-supplemented media for 5 days. Error bars represent standard deviation (*n* = 6) for three biological replicates. An asterisk indicates a significant difference from the wild type (*Col-0*), determined by the Student’s t test (*P* < 0.05)

The ALS regulatory subunits have been shown to have two functional roles in the holoenzyme complex: they act to stabilize the ALS complex thus contributing to the enhancement of ALS catalytic activity, and are also responsible for BCAA-mediated end-point inhibition of ALS catalytic activity, most likely via the highly-conserved ACT domain [5, 6]. To investigate whether the two regulatory subunits may have different effects on ALS activity, we examined the axenic growth of *aip* knockout mutants in media supplemented with exogenous BCAAs. The analyses revealed that the *aip3-1* line was relatively insensitive to primary root inhibition by Val and Ile in comparison to the *Col*-0 wild-type (Fig. 5 & Additional file 1: Figure S4). Indeed, *aip3-1* exhibited enhanced growth in comparison to the wild type and *aip1-2* in media supplemented with 250 μM and 500 μM Val. However, *aip1-2* exhibited a pattern of primary root inhibition similar to wild-type plants. Notably, there were no observable differences in primary root elongation inhibition between the two *aip* mutants and *Col*-0 among seedlings germinated on Leu-supplemented media (Fig. 5 & Additional file 1: Figure S4). These root elongation-inhibition patterns were reproducible across three independent experiments, and suggest that AIP3 plays a principal role in Val- and Ile-mediated feedback inhibition in Arabidopsis roots.

**Fig. 6:**
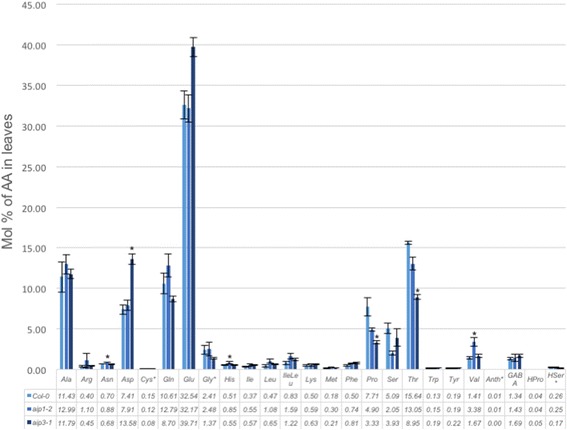
Protein amino acid profiles of two *aip1-2* and *aip3* mutants. Concentration of 20 free protein amino acids (nmol mg^−1^) in *Col*-0*, aip1-2* and *aip3-1* in leaves. Average values ± SD of three biological replicates are given. An asterisk indicates a significant difference from the wild type, determined by the Student’s t test (*P* < 0.05)

Given our finding that the root growth of *aip3-1* mutants was resistant to Val and Ile inhibition, we reasoned this might be the consequence of a loss of feedback inhibition by BCAAs on the biosynthesis pathway. If true, one would hypothesize that mutant *aip1* and *aip3* plants would exhibit elevated steady-state levels of BCAAs in plant tissues. To investigate this possibility, we measured the abundance of free amino acids in leaf tissues from 28-day-old *aip1-2* and *aip3-1* plant genetic lines using an LC-MS/MS-based approach (Fig. 6). The results revealed a modest increase in the abundance of Val and Leu in the leaves of *aip3-1* background, and an approximate two-fold increase in the abundance of Val and Leu in the *aip1-2* mutant. Taken together, the results suggest that, while *AIP1* and *AIP3* genes have overlapping functions, they have also acquired distinct roles in the regulation of BCAA synthesis.

To assess whether the ALS regulatory subunits might also contribute to functions other than the canonical BCAA synthesis pathway, we examined the response of *aip1-2* and *aip3-1* mutant plants to a panel of hormonal and abiotic stress treatments. In one such study involving osmotic stress, we observed no difference in the NaCl-mediated inhibition of root elongation among the two *aip1* and *aip3* knockout mutants versus the wild-type *Col-*0 (Additional file 1: Figure S5). However, *aip3-1* mutant seedlings exhibited a progressively more severe chlorotic phenotype when transferred to media supplemented with increasing concentrations of NaCl (Additional file 1: Figure S5). This chlorotic phenotype was not observed on KCl-supplemented media or on osmotic stress-inducing media supplemented with iso-molar concentrations of sorbitol or mannitol, suggesting that the phenotype involves some unknown aspect of sodium-ion (Na^+^) homeostasis.

## Discussion

While the ALS enzyme is found in almost all autotrophic organisms, and has been extensively studied in the context of BCAA synthesis, relatively little is known about how this enzymatic pathway is regulated in plants. Despite the presence of multiple feedback regulatory subunits, the functional contribution of each to BCAA biosynthesis is not well understood. In this study, we have undertaken to investigate the functional contribution of each one of these regulatory subunits via a set of complimentary genetic and biochemical approaches.

Our Y2H screen identified eight novel polypeptides capable of interacting with the ALS catalytic subunit. Further genetic and biochemical studies revealed two of these proteins can contribute to the regulation of BCAA synthesis. Further studies are required to assess whether the remaining six proteins also contribute to BCAA homeostasis. Interestingly, the subcellular localization of the remaining six proteins have been shown to include the chloroplast, either through biochemical demonstration or computational prediction, suggesting these proteins are likely true ALS interacting partners.

The identification of multiple interacting partners for ALS in Y2H screens may suggest that the ALS holoenzyme could be involved in, or regulated by, mechanisms unrelated to BCAA synthesis, or that the novel interacting partners identified are involved in BCAA synthesis via a yet-to-be-determined regulatory mechanism. Furthermore, experimental evidence provided to date has shown that enzymes involved in the metabolism of BCAA can participate in the formation of multi-enzyme complexes involving proteins outside of the BCAA synthesis pathway. There is also a possibility that they possess protective roles, similar to those described for RIDA proteins. RIDA proteins have been shown to protect plants during BCAA biosynthesis by hydrolysing short-lived reactive enamine/imine intermediates [21]. Vitamin B6 (pyridoxal 5′-phosphate) is an essential cofactor of branched-chain amino acid transaminase (BCAT) [22, 23]. BCAT proteins are responsible for the last step of BCAA synthesis in plastids and can also initiate the degradation of BCAAs in mitochondria. Plants can synthesize this vitamin de novo via two enzymes, pyridoxine synthase1 (PDX1) and PDX2. Interestingly, PDX1 was identified as an interacting partner for ALS in our Y2H screen. If verified and PDX1 or ALS activities are effected via this interaction, it is possible that crosstalk between the first step and the last step of BCAA synthesis exists, as well as the likelihood of interplay between BCAA and B6 synthesis pathways.

The expression of two ALS regulatory subunit-encoding genes in Arabidopsis raised the question whether each contributes differentially to BCAA synthesis. While both proteins similarly interact with the ALS catalytic subunit as evidenced by Y2H studies, the steady-state abundance of their transcripts was found to be markedly different in select tissues, suggesting a potential differential contribution to BCAA synthesis. We initially investigated root elongation in seedlings of both *aip1-2* and *aip3-1* genetic backgrounds in the presence of various BCAAs, as an indirect measure of sensitivity of ALS enzyme to their end products. While ALS inhibition by end products results in stunted root elongation in both wild-type and *aip1-1* plants, the *aip3-1* mutant line was insensitive to elevated concentrations of both Val and Ile. This differential root elongation phenotype in aip*3-1* versus *aip1-2* mutant backgrounds on Val- and Ile-supplemented media suggests that AIP3 may play a greater role during ALS feedback inhibition during root growth notwithstanding the relative lower transcript abundance of *AIP3* in most tissues. Notably, no difference in growth inhibition between the two *aip* mutant lines was apparent on Leu-supplemented media, suggesting that the structural variation evident in the second ACT repeat of AIP1 versus AIP3 may constitute the site of the Val/Ile-binding and inhibition. Future studies might assess whether the catalytic activity of the holoenzyme in complex with AIP3 and BCAAs as cofactors exhibits a greater sensitivity to Val and Ile compared to the holoenzyme in complex with AIP1.

Whereas all three C-terminal YFP-fusions to ALS, AIP1, and AIP3 were found to localize to chloroplasts, N-terminal YFP-fusions to two AIP proteins resulted in their localization to peroxisomes. N-terminal tagging of ALS resulted in the exclusion of the ALS catalytic subunit from the chloroplast and localization to the cytoplasm, with no evidence for co-localization with a known peroxisomal marker protein [15]. Thus, while fusion proteins involving the ALS catalytic subunit are apparently localized exclusively to the chloroplast, fusions involving AIP1 and AIP3 exhibit a more diverse localization pattern. These findings suggest that both AIP1 and AIP3 regulatory subunits have acquired functional attributes independent of the ALS holoenzyme. Interestingly, a similar phenomenon has been described for several other proteins in Arabidopsis and other plant species [19]. While BCAA synthesis has been localized chiefly to plastids, the degradation of BCAAs have been hypothesized to occur mainly in mitochondria and peroxisomes [24]. Given that both ALS regulatory subunits can bind BCAAs even in the absence of the ALS catalytic subunit, it is possible that AIP1 and AIP3 can contribute to binding and degradation of BCAAs in peroxisomes via a yet-to-be determined mechanism.

The present study revealed a several-fold increase in *AIP3* transcript abundance in reproductive tissues compared to all other tissues examined. The finding that 30–40% of ovules were aborted in *aip1-2/AIP1 aip3-1/aip3-1* genetic backgrounds suggests that AIP3 plays a significant role in reproductive tissue development and/or maintenance, noting that the wild-type *AIP1* allele effectively rescued the silique seed phenotype in *AIP1/AIP1 aip3-1/aip3-1* genetic backgrounds. Thus, the aborted ovule phenotype was not likely the result of double mutant allelic segregation, since the aborted seed phenotype was not observed in the *aip1-2/aip1-2 aip3-1/AIP3* mutant backgrounds. The *aip* double mutants failed to develop adult organs, becoming chlorotic and dying at an early stage—possibly after the nutrients stored in the proto-organs were exhausted. Interestingly, casamino acid- and BCAA-supplemented media failed to rescue the severe developmental phenotype exhibited by *aip* double mutants (data not shown). This result was surprising considering that ALS regulatory subunit knockouts in bacteria and yeast are non-lethal [25].

The results presented in this study suggest that AIP polypeptides contribute other essential as-yet unknown functions in development that are unrelated to BCAA homeostasis. While beyond the scope of this study, future work might investigate whether the aberrant abnormality lies in pollen development, pollen tube formation, megaspore development, or later aspects of zygote development among segregating progeny from an *aip1-2/AIP1 aip3-1/aip3-1* X *Col*-0 reciprocal crosses.

Our finding that pre-elongated roots of *aip3-1* mutants exhibit a chlorotic phenotype on NaCl-supplemented media is the second such report indicating that genetic alteration of BCAA metabolism results in a corresponding loss of salt homeostasis [26]. As described here, the chlorotic phenotype of plants homozygous for the *aip3-1* allele is restricted to pre-elongated roots, since seeds germinated directly on salt-supplemented media failed to exhibit the phenotype. Moreover, the observed salt sensitivity was restricted to Na^+^ salts, since supplementation of media with either KCl or inert osmotic agents (mannitol or sorbitol) failed to elicit the chlorotic phenotype (Additional file 1: Figure S5). Taken together, our results suggest that *aip3-1* mutants are altered in some unknown aspect of Na^+^ homeostasis. Others have suggested that elevated levels of BCAAs in perturbed genetic systems may act to protect plants to general osmotic stress [26, 27]. However, our finding that the observed salt-induced phenotype is Na^+^ specific and unrelated to a general osmotic stress agent response suggests a more direct connection between BCAA metabolism and Na^+^ homeostasis. Indeed, others have shown that genetic oblation of a pyridoxal kinase gene resulting in loss of biosynthesis of pyridoxal 5′-phosphate (Vitamin B6; PLP)—an obligatory cofactor in the catabolism of BCAAs—results in a salt-over-sensitive phenotype in roots of Arabidopsis [28, 29]. It is tempting to speculate that an elevation in the steady state levels of BCAAs through genetic manipulation leads to a concomitant alteration of PLP, in turn resulting in an alteration in Na^+^ homeostasis.

## Conclusion

The results described here suggest that current notions of how the BCAA biosynthetic pathway is regulated may be oversimplified. Based on our results, we propose a revised BCAA regulatory model that incorporates an added layer of complexity for the regulation of BCAA synthesis via two competing regulatory subunits, as well as intermolecular cross-talk involving other pathways important for reproductive development and Na^+^ homeostasis. The differential localization of the ALS catalytic subunit with AIPs suggests additional unknown sub-functions as revealed by subcellular location to compartments (e.g., peroxisomes) other than the chloroplast. Studies of patterning and development in single- and multiple-mutant mutant genetic backgrounds suggests that unknown AIP homologous functions not directly associated with BCAA synthesis are required to sustain early development and viability in Arabidopsis.

## Methods

### Plant materials and growth conditions

Transgenic *Arabidopsis thaliana* ecotype *Col-*0 lines identified as *aip1-2* and *aip3-1* (Additional file 1: Table S1) were drawn from the Salk T-DNA insertion mutagenesis resource [20] and subsequently used in this study. The wild-type *A. thaliana* isogenic line CS7000 (Arabidopsis Biological Resource Center, Ohio State University) was used for comparative analyses.

For germination studies, 100–200 seeds from transgenic and wild type lines were surface-sterilized in 200 μL 50% ethanol followed by a 10-min incubation in 200 μL of 50% bleach and 0.3% (*w/v*) SDS solution. Seeds were washed four times in autoclaved high purity water and plated on 10-cm Petri dishes with 0.5× MS [30] media containing 1% (*w/v*) sucrose and 0.6% (*w*/*v*) agar at pH 5.7, unless otherwise stated. Plates were sealed with 3 M Micropore™ porous tape.

Seeds were stratified at 4 °C for 48 h in dark then transferred to growth chambers set at 100% humidity, 21 °C and a 24-h 200 μM m^−2^ s^−1^ photon light cycle for germination and growth. Once seedlings reached the 6-true-leaf stage they were transferred to Greenworld™ original formula soil in 64-cm^2^ pots, 4 per unit. Plants were grown under a 16/8-h light/dark cycle and watered with tap water daily. Upon maturation, browned siliques were collected and stored in 1.5-mL microcentrifuge tubes. As the *aip1-2* and *aip3-1* mutants grew, phenotypic characterization was performed and photographed by a Canon PowerShot™ SX120 IS camera.

*N. benthamiana* seeds were acquired from Fathey Sarhan Lab (University of Québec at Montréal). Agrobacterium strain (AGL-1) [31] was used for both transgenic generation and transient expression in *N. benthamiana* leaves.

### Root elongation assay

Seeds from transgenic and wild type lines were prepared and platted on 0.5× MS media. After stratification, plates were oriented vertically in 24-h light for 5 days. Seedlings were subsequently transferred to hormone-, BCAA-, metabolite- or herbicide-supplemented 0.5× MS media containing 3% (*w/v*) sucrose and 0.6% (*w/v*) agar to promote root growth. Root tips were marked on the base of the plate and the seedlings were grown vertically in constant light for an additional 5 days. Root elongation was measured and compared to growth on standard 0.5× MS media containing 3% (*w/v*) sucrose and 0.6% (*w/v*) agar plate.

### Transgenic selection on Basta™-supplemented media

Seeds from plants subjected to *Agrobacterium*-mediated floral dip transformation [32] were surface-sterilized as aforementioned and sown on 0.5× MS media supplemented with 50 μM Basta™ (Liberty 200LN). Seeds were stratified for 2 days, grown in 24-h light for 12-h and then grown in dark for 48 h to elongate seedling hypocotyls. After an additional 48 h of growth in light, transgenic seedlings with green hypocotyls were selected and the presence of the transgene was confirmed by PCR or confocal fluorescence microscopy.

### Genotyping of Arabidopsis mutants by PCR

PCR analysis was used to confirm *aip1-1 aip1-2* and *aip3-1* genotypes. The LB and RB primers used are listed in Additional file 1: Table S1, along with the T-DNA-specific LB primer, LBb1.3.

### Real-time-PCR analysis

Whole seedlings (0.1 g) were flash-frozen in liquid nitrogen and ground using a mortar and pestle. RNA was extracted and DNA degraded using the Qiagen RNeasy Plant Mini Kit as per the manufacturer’s protocol. RNA (5 μg) was used for the reverse transcriptase reaction using Maxima First Strand cDNA Synthesis Kit (Thermo Fisher Scientific Inc.) for RT-qPCR as per manufacturer’s protocol. LB and RB primers specific to *AIP1*, *AIP3* and *ACT2* cDNA are listed in Additional file 1: Table S2. *ACT2* was used as an internal control for relative transcript abundance. Real-time-PCR reactions were cycled using an Applied Biosystems ViiA 7™ Real-Time PCR System (Life Technologies). Data analysis was performed as described earlier [33].

### Confocal fluorescence microscopy

Select mutant coding regions were cloned into pEARLEYGATE104 or pEARLEYGATE101 vectors for expression as N-terminal or C-terminal YFP-fusions, respectively. *Agrobacterium* harboring the indicated expression constructs were infiltrated into *N. benthamiana* leaves along with p19, as previously described [33]. Transfected *N. benthamiana* abaxial leaf tissue expressing the YFP-fusion proteins were imaged using a Leica DMI6000 epifluoresence microscope fitted with a GFP or YFP fluorescent filter cube or Olympus Model FV1000 point-scanning/point-detection laser scanning confocal microscope. Cyan fluorescent protein (CFP), yellow fluorescent protein (YFP), and propidium iodide (PI) were excited by using 440, 512, and 543 nm laser lines, respectively. When using multiple fluorophores simultaneously, images were acquired sequentially to reduce excitation and emission overlaps. Olympus water immersion PLAPO60XWLSM (NA 1.0) and UPLSAPO 20× (NA 0.75) objectives were employed. Image acquisition was conducted at a resolution of 512 × 512 pixels, with a scan rate of 10 ms per pixel. Olympus FLUOVIEW v1.5 software was used for image acquisition and the export of TIFF files. Figures were assembled using GIMP 2.0 as described earlier [33].

### Free amino acid analysis

Approximately, 20 mg of leaf tissue from 28-day old *Col*-0 wild-type, *aip1-2* and *aip3-1* plants were snap frozen in liquid nitrogen. Free amino acid extraction and analysis by HPLC-MS/MS was performed as described [34]. The analysis shown is representative of three independent biological and sample replicas.

## Additional file

Additional file 1: Figure S1. Alignment of Arabidopsis, yeast, and *E. coli* ALS regulatory subunits. Sequence alignment is based on the deduced primary amino acid sequence. Black and gray boxes denote conserved and semi conserved amino acids respectively. **Figure S2.** Alignment of Arabidopsis AIP1 and AIP3, the ALS regulatory subunits. Sequence alignment is based on the deduced primary amino acid sequence. Black and gray boxes denote conserved and semi conserved amino acids respectively. **Figure S3.** (A) Siliques of wild type and *aip* mutant plants. Siliques of *Col*-0 (right) and single wild type *AIP* allele mutant lines under a light box. Vacant spaces are considered to be aborted seeds. Only *AIP1/aip1-2 aip3-1/aip3-1* mutants exhibit a reduced fecundity phenotype. (B) Measurement of seed count in mature siliques of wild type and *aip* knockout mutants. Fecundity of single wild type *AIP* allele and single *aip* knockout genetic lines was measured by seed count in mature siliques. The error bars represent standard deviation (*n* = 8) and two biological replicates. **Figure S4.** 5-day-old seedlings were transferred from non-supplemented media (0.5× MS) to supplemented media as labelled. Primary root inhibition was measured in *aip* mutants and wild-type seedlings on BCAA-supplemented media. **Figure S5.** Primary root inhibition in *aip* mutant seedlings in various supplemented media. Percent primary root inhibition in 5-day-old seedlings grown on sodium chloride- and potassium chloride-supplemented media, and osmotic stress-inducing media with mannitol or sorbitol for an additional 5 days. The error bars represent standard deviation (*n* = 6) and two biological replicates. **Table S1.** qRT-PCR primers used in this study. **Table S2.** Genotyping primers used in this study. (DOCX 15054 kb)

## Abbreviations

0.5X MS: 0.5X Murashige and Skoog medium
ACT domain: Aspartate kinase, chorismate mutase, TyrA domain
AHAS: Acetohydroxyacid synthase
AIP: ALS-Interacting Protein
ALS: Acetolactate synthase
BCAA: Branched-chain amino acid
BCAT: Branched-chain amino acid transaminase
BiFC: Bimolecular fluorescence complementation
cDNA: Complementary DNA
CPN10: Chloroplast Chapteronin 10
GFP: Green fluorescent protein
GUS: β-glucuronidase
Ile: Isoleucine
Leu: Leucine
MS/MS: Tandem mass spectrometry
MS: Mass spectrometry
PDX: Pyridoxine synthase
PDX1.1: Pyridoxine Biosynthesis 1.1
PLP: Pyridoxal phosphate
PTS: Peroxisomal targeting sequence
Px-CFP: Peroxisomal cyan fluorescent protein marker
qRT-PCR: Quantitative reverse transcriptase polymerase chain reaction
RING: Really Interesting New Gene
S-K-Y: Serine-lysine-tyrosine
S-T-Y: Serine-threonine-tyrosine
T-DNA: Transfer DNA
Val: Valine
Y2H: Yeast two-hybrid
YFP: Yellow fluorescent protein

## References

[1] Umbarger HE, Brown B (1958). Isoleucine and valine metabolism in *Escherichia coli*. VIII. The formation of acetolactate. J Biol Chem.

[2] Zhou Q, Liu W, Zhang Y, Liu KK (2007). Action mechanisms of acetolactate synthase-inhibiting herbicides. Pestic Biochem Physiol.

[3] Chen H, Saksa K, Zhao F, Qiu J, Xiong L (2010). Genetic analysis of pathway regulation for enhancing branched-chain amino acid biosynthesis in plants. Plant J.

[4] Haughn GW, Somerville CR (1990). A mutation causing Imidazolinone resistance maps to the Csr1 locus of *Arabidopsis thaliana*. Plant Physiol.

[5] Lee YT, Duggleby RG (2001). Identification of the regulatory subunit of *Arabidopsis thaliana* acetohydroxyacid synthase and reconstitution with its catalytic subunit. Biochemistry.

[6] Lee YT, Duggleby RG (2002). Regulatory interactions in *Arabidopsis thaliana* acetohydroxyacid synthase. FEBS Lett.

[7] Chipman D, Barak Z, Schloss JV (1998). Biosynthesis of 2-aceto-2-hydroxy acids: acetolactate synthases and acetohydroxyacid synthases. Biochim Biophys Acta.

[8] Singh B, Szamosi I, Hand JM, Misra R (1992). Arabidopsis Acetohydroxyacid Synthase expressed in *Escherichia coli* is insensitive to the feedback inhibitors. Plant Physiol.

[9] Miflin BJ (1971). Cooperative feedback control of barley acetohydroxyacid synthetase by leucine, isoleucine, and valine. Arch Biochem Biophys.

[10] Hershey HP, Schwartz LJ, Gale JP, Abell LM (1999). Cloning and functional expression of the small subunit of acetolactate synthase from *Nicotiana plumbaginifolia*. Plant Mol Biol.

[11] Ober D (2005). Seeing double: gene duplication and diversification in plant secondary metabolism. Trends Plant Sci.

[12] Vyazmensky M, Sella C, Barak Z, Chipman DM (1996). Isolation and characterization of subunits of acetohydroxy acid synthase isozyme III and reconstitution of the holoenzyme. Biochemistry.

[13] Grimminger H, Umbarger HE (1979). Acetohydroxy acid synthase I of *Escherichia coli*: purification and properties. J Bacteriol.

[14] Hooper CM, Tanz SK, Castleden IR, Vacher MA, Small ID, Millar AH (2014). SUBAcon: a consensus algorithm for unifying the subcellular localization data of the Arabidopsis proteome. Bioinformatics (Oxford, England).

[15] Nelson BK, Cai X, Nebenfuhr A (2007). A multicolored set of in vivo organelle markers for co-localization studies in Arabidopsis and other plants. Plant J.

[16] Mazur BJ, Chui CF, Smith JK (1987). Isolation and characterization of plant genes coding for acetolactate synthase, the target enzyme for two classes of herbicides. Plant Physiol.

[17] Emanuelsson O, Elofsson A, von Heijne G, Cristobal S (2003). In silico prediction of the peroxisomal proteome in fungi, plants and animals. J Mol Biol.

[18] Meyer T, Holscher C, Schwoppe C, von Schaewen A (2011). Alternative targeting of Arabidopsis plastidic glucose-6-phosphate dehydrogenase G6PD1 involves cysteine-dependent interaction with G6PD4 in the cytosol. Plant J.

[19] Carrie C, Whelan J (2013). Widespread dual targeting of proteins in land plants: when, where, how and why. Plant Signal Behav.

[20] Alonso JM, Stepanova AN, Leisse TJ, Kim CJ, Chen H, Shinn P, Stevenson DK, Zimmerman J, Barajas P, Cheuk R (2003). Genome-wide insertional mutagenesis of *Arabidopsis thaliana*. Science (New York, NY).

[21] Niehaus TD, Nguyen TN, Gidda SK, ElBadawi-Sidhu M, Lambrecht JA, McCarty DR, Downs DM, Cooper AJ, Fiehn O, Mullen RT (2014). Arabidopsis and maize RidA proteins preempt reactive enamine/imine damage to branched-chain amino acid biosynthesis in plastids. Plant Cell.

[22] Diebold R, Schuster J, Daschner K, Binder S (2002). The branched-chain amino acid transaminase gene family in Arabidopsis encodes plastid and mitochondrial proteins. Plant Physiol.

[23] Amorim Franco TM, Hegde S, Blanchard JS (2016). Chemical mechanism of the branched-chain Aminotransferase IlvE from mycobacterium tuberculosis. Biochemistry.

[24] Binder S (2010). Branched-chain amino acid metabolism in *Arabidopsis thaliana*. Arabidopsis Book / American Society of Plant Biologists.

[25] Lu M-F, Umbarger H (1987). Effects of deletion and insertion mutations in the ilvM gene of *Escherichia coli*. J Bacteriol.

[26] Zhang C, Pang Q, Jiang L, Wang S, Yan X, Chen S, He Y (2015). Dihydroxyacid dehydratase is important for gametophyte development and disruption causes increased susceptibility to salinity stress in Arabidopsis. J Exp Bot.

[27] Joshi V, Joung JG, Fei ZJ, Jander G (2010). Interdependence of threonine, methionine and isoleucine metabolism in plants: accumulation and transcriptional regulation under abiotic stress. Amino Acids.

[28] Shi H, Zhu JK (2002). SOS4, a pyridoxal kinase gene, is required for root hair development in Arabidopsis. Plant Physiol.

[29] Shi H, Xiong L, Stevenson B, Lu T, Zhu JK (2002). The Arabidopsis salt overly sensitive 4 mutants uncover a critical role for vitamin B6 in plant salt tolerance. Plant Cell.

[30] Murashige T, Skoog F (1962). A revised medium for rapid growth and bioassays with tobacco tissue cultures. Physiol Plant.

[31] Lazo GR, Stein PA, Ludwig RA (1991). A DNA transformation-competent Arabidopsis genomic library in Agrobacterium. Bio/technology (Nature Publishing Company).

[32] Clough SJ, Bent AF (1998). Floral dip: a simplified method for Agrobacterium-mediated transformation Of*arabidopsis thaliana*. Plant J.

[33] Dezfulian MH, Soulliere DM, Dhaliwal RK, Sareen M, Crosby WL (2012). The *SKP1-like* Gene family of Arabidopsis exhibits a high degree of differential Gene expression and Gene product interaction during development. PLoS One.

[34] Gu L, Jones AD, Last RL (2007). LC-MS/MS assay for protein amino acids and metabolically related compounds for large-scale screening of metabolic phenotypes. Anal Chem.

