# Supplementary material for: Acetolactate synthase regulatory subunits play divergent and overlapping roles in branched-chain amino acid synthesis and Arabidopsis development
Source: BMC Plant Biol. 2017 Apr 7;17:71. doi: 10.1186/s12870-017-1022-6 (PMC5384131; doi:10.1186/s12870-017-1022-6)
Supplement: Additional file 1: Figure S1. — Alignment of Arabidopsis, yeast, and E. coli ALS regulatory subunits. Sequence alignment is based on the deduced primary amino acid sequence. Black and gray boxes denote conserved and semi conserved amino acids respectively. Figure S2. Alignment of Arabidopsis AIP1 and AIP3, the ALS regulatory subunits. Sequence alignment is based on the deduced primary amino acid sequence. Black and gray boxes denote conserved and semi conserved amino acids respectively. Figure S3. (A) Siliques of wild type and aip mutant plants. Siliques of Col-0 (right) and single wild type AIP allele mutant lines under a light box. Vacant spaces are considered to be aborted seeds. Only AIP1/aip1-2 aip3-1/aip3-1 mutants exhibit a reduced fecundity phenotype. (B) Measurement of seed count in mature siliques of wild type and aip knockout mutants. Fecundity of single wild type AIP allele and single aip knockout genetic lines was measured by seed count in mature siliques. The error bars represent standard deviation (n = 8) and two biological replicates. Figure S4. 5-day-old seedlings were transferred from non-supplemented media (0.5× MS) to supplemented media as labelled. Primary root inhibition was measured in aip mutants and wild-type seedlings on BCAA-supplemented media. Figure S5. Primary root inhibition in aip mutant seedlings in various supplemented media. Percent primary root inhibition in 5-day-old seedlings grown on sodium chloride- and potassium chloride-supplemented media, and osmotic stress-inducing media with mannitol or sorbitol for an additional 5 days. The error bars represent standard deviation (n = 6) and two biological replicates. Table S1. qRT-PCR primers used in this study. Table S2. Genotyping primers used in this study. (DOCX 15054 kb) [file 12870_2017_1022_MOESM1_ESM.docx]

**Acetolactate** **synthase regulatory subunits play divergent and overlapping roles in branched-chain amino acid synthesis and Arabidopsis development**

Mohammad H. Dezfulian^1,a^, Curtis Foreman^1^, Espanta Jalili^1^, Mrinal Pal^1^, Rajdeep K. Dhaliwal^1^, Don Karl A. Roberto^1^, Kathleen M. Imre^2^, Susanne E. Kohalmi^3^, William L. Crosby^1,*^

^1^ Department of Biological Sciences, University of Windsor, Windsor, Ontario, Canada

^2^ Department of Biochemistry and Molecular Biology, Michigan State University, Lansing, Michigan, USA

^3^ Department of Biology, University of Western Ontario, London, ON, Canada

^a^ Present address: Department of Genetics, Harvard Medical School, Boston, MA 02115

^*^Corresponding author: [bcrosby@uwindsor.ca](mailto:bcrosby@uwindsor.ca)


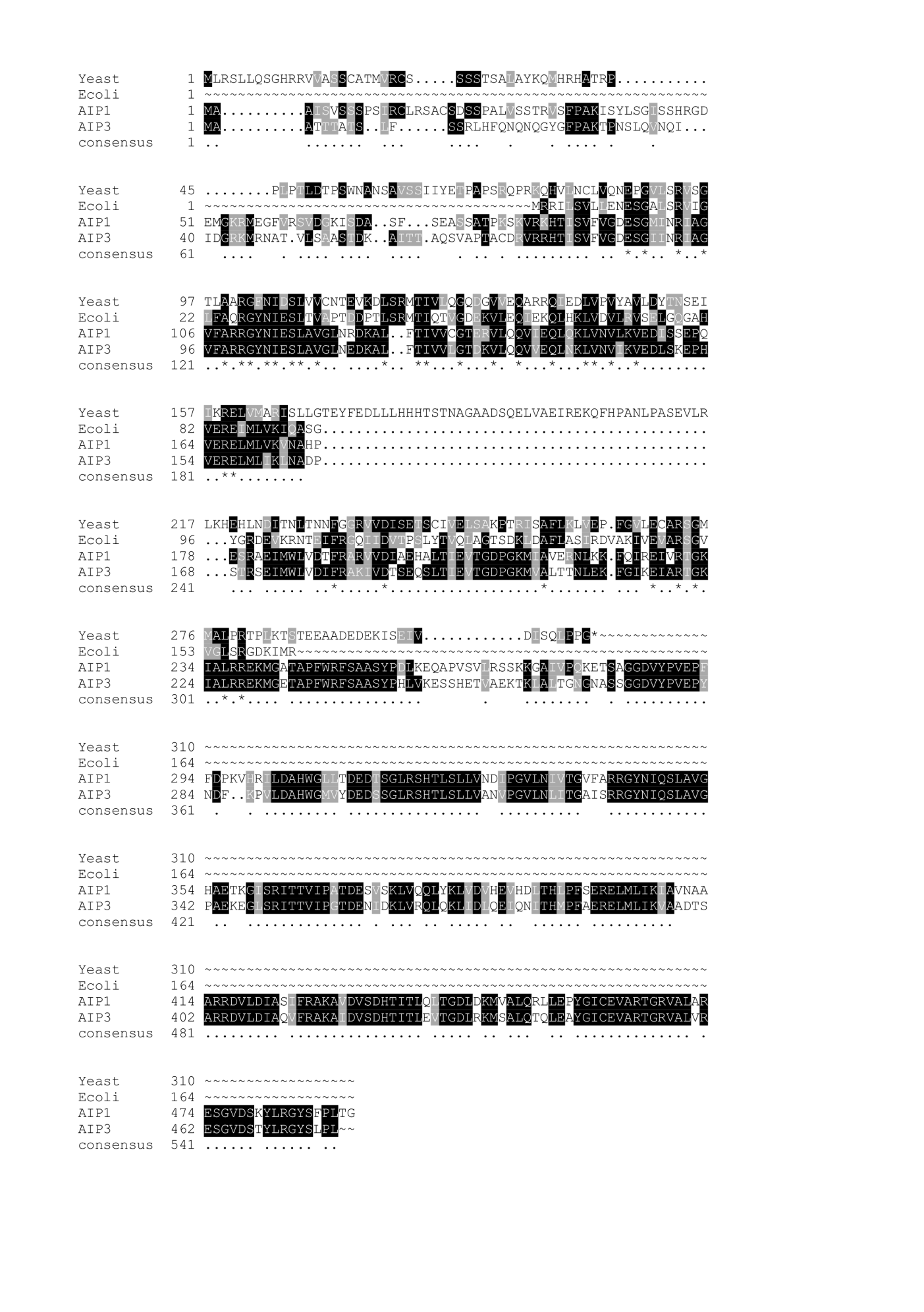


**Figure S1.** Alignment of Arabidopsis, yeast, and *E. coli* ALS regulatory subunits. Sequence alignment is based on the deduced primary amino acid sequence. Black and gray boxes denote conserved and semi conserved amino acids respectively.

**
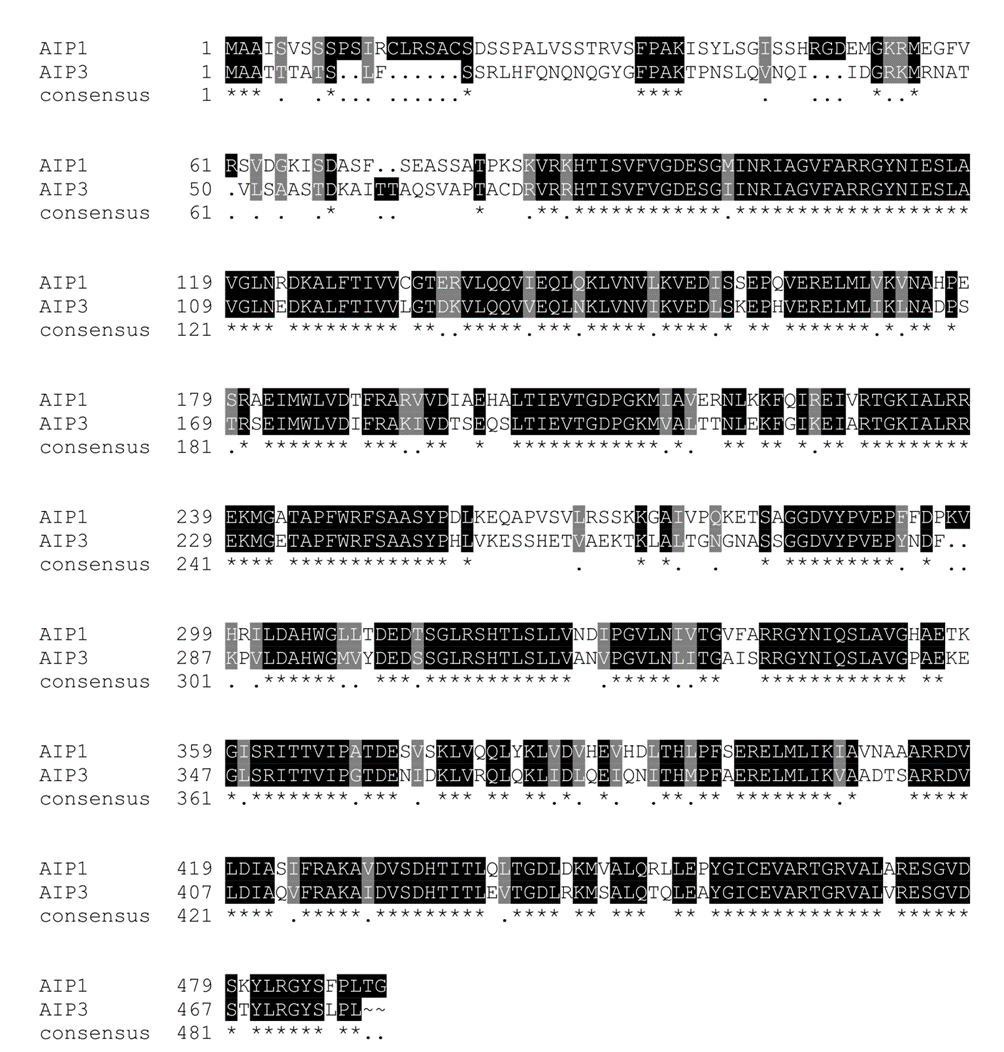
**

**Figure S2.** Alignment of Arabidopsis AIP1 and AIP3, the ALS regulatory subunits. Sequence alignment is based on the deduced primary amino acid sequence. Black and gray boxes denote conserved and semi conserved amino acids respectively.

A

B

**Figure S3.** **(A)** Siliques of wild type and *aip* mutant plants. Siliques of *Col*-0 (right) and single wild type *AIP* allele mutant lines under a light box. Vacant spaces are considered to be aborted seeds. Only *AIP1/aip1-2 aip3-1/aip3-1* mutants exhibit a reduced fecundity phenotype. (**B)** Measurement of seed count in mature siliques of wild type and *aip* knockout mutants. Fecundity of single wild type *AIP* allele and single *aip* knockout genetic lines was measured by seed count in mature siliques. The error bars represent standard deviation (*n*=8) and two biological replicates.

**Figure S4.** 5-day-old seedlings were transferred from non-supplemented media (0.5x MS) to supplemented media as labelled. Primary root inhibition was measured in *aip* mutants and wild-type seedlings on BCAA-supplemented media.

**Figure S5.** Primary root inhibition in *aip* mutant seedlings in various supplemented media. Percent primary root inhibition in 5-day-old seedlings grown on sodium chloride- and potassium chloride-supplemented media, and osmotic stress-inducing media with mannitol or sorbitol for an additional 5 days. The error bars represent standard deviation (*n*=6) and two biological replicates.

**Table S1. qRT-PCR primers used in this study.**

| **Gene** | **Atg Identifier** | **Primer Sequence** |
| --- | --- | --- |
| *ACT 2* | AT3G18780 | LP: TCCTCTCCGCTTTGAATTGTCTCG  RP: GGATGGCATGAGGAAGAGAGAAACC |
| *AIP1* | AT2G31810 | LP: CGGAACATGCATTGACTATCGAGGT  RP: GCGTTCACGGCAATCTTAATCAGC |
| *AIP3* | AT5G16290 | LP: TCCGACGGCTTGTGATAGAGTAAGG  RP: GGACCGGTTTGAAATCGTTATAGGG |

**Table S2. Genotyping primers used in this study.**

| **Plant Lines** | **Atg Identifier** | **Gene** | **Genotyping Primers** |
| --- | --- | --- | --- |
| *aip1-1* (SALK_053116) | AT3G18780 | AIP1 | LP: CAAGCCACAAATAGAAGCTGC  RP: CATCGACGCTTCTAACGAATC |
| *aip1-2* (SALK_096207) | AT2G31810 | AIP1 | LP: TAAGGTCGTCAACAACCGAAC  RP: CAGAAGTCTGAAACCCACAGG |
| *aip3-1* (SALK_020021) | AT5G16290 | AIP3 | LP: TTGCCGTAGGATTGAATGAAG  RP: TGTCCTGAGATTCACCAAA |
